# Revisiting Epithelial Carcinogenesis

**DOI:** 10.3390/ijms23137437

**Published:** 2022-07-04

**Authors:** Luis Fernando Méndez-López

**Affiliations:** Centro de Investigación en Nutrición y Salud Pública, Facultad de Salud Pública y Nutrición, Universidad Autonoma de Nuevo Leon, Monterrey 66460, Mexico; luis.mendezlop@uanl.edu.mx; Tel.: +52-81-1340-4890 (ext. 1340)

**Keywords:** senescence, EMT, NF-κB, inflammation, epigenetics, aging, TNBC, NAFLD

## Abstract

The origin of cancer remains one of the most important enigmas in modern biology. This paper presents a hypothesis for the origin of carcinomas in which cellular aging and inflammation enable the recovery of cellular plasticity, which may ultimately result in cancer. The hypothesis describes carcinogenesis as the result of the dedifferentiation undergone by epithelial cells in hyperplasia due to replicative senescence towards a mesenchymal cell state with potentially cancerous behavior. In support of this hypothesis, the molecular, cellular, and histopathological evidence was critically reviewed and reinterpreted when necessary to postulate a plausible generic series of mechanisms for the origin and progression of carcinomas. In addition, the implications of this theoretical framework for the current strategies of cancer treatment are discussed considering recent evidence of the molecular events underlying the epigenetic switches involved in the resistance of breast carcinomas. The hypothesis also proposes an epigenetic landscape for their progression and a potential mechanism for restraining the degree of dedifferentiation and malignant behavior. In addition, the manuscript revisits the gradual degeneration of the nonalcoholic fatty liver disease to propose an integrative generalized mechanistic explanation for the involution and carcinogenesis of tissues associated with aging. The presented hypothesis might serve to understand and structure new findings into a more encompassing view of the genesis of degenerative diseases and may inspire novel approaches for their study and therapy.

## 1. Introduction

Cancer remains the second leading cause of death worldwide [1] despite the enormous amounts of research and financial support devoted to finding the cure [2]. In essence, the development of effective therapeutic strategies remains limited by the incomplete understanding of the underlying causes of its emergence and progression [3,4]. The somatic mutation theory continues as the prevailing paradigm and assumes that cancer is a genetic disease in which the alterations in the genetic material account for the cancerous phenotype [5]. Therefore, the rationale has been to kill cancer cells, usually via DNA poisons in combination with agents that target the molecular alterations associated with the aberrant genetic traits [6]. Strikingly, most antineoplastic agents and radiotherapy induce cancer in humans [7], and the initial favorable response is temporal and followed by tumor progression despite correct and complete administration of the protocols [8,9].

The contribution of aging and inflammation to cancer development is mainly linked with mutations since the traditional view conveys that carcinogenesis is a multistep process related to genetic or chromatin damage [10]. Hence, the exponential rise in carcinoma incidence associated with age, especially in patients suffering from chronic inflammation [11], has been interpreted as the consequence of an increased likelihood to acquire defects in the genetic material of epithelial cells. Under the somatic mutation theory, telomere attrition in aged cells and the production of oxidative stress by immune cells favor the attainment of malignant features by mutagenesis and chromosomal instability with the potential to evolve into cancerous behavior [12,13,14]. Furthermore, the accumulation of senescent cells associated with aging is considered to contribute to structural and endocrine changes that may also promote carcinogenesis of the affected tissues [15,16,17]. In this regard, they produce cytokines, chemokines, growth factors, and proteases that attract and activate immune cells. In turn, the burden of inflammation and tissue disruption promotes cell proliferation and the epithelial-to-mesenchymal transition (EMT) [18,19]. This process has been linked with cancer invasion [20], metastasis [21], and the generation of cells with features of mesenchymal stem cells [22] or cancer stem cells [19] that are drug-resistant [23], enabled for immune system evasion, and from which [24] relapses may originate [25]. Overall, the conventional view suggests that, in the first instance, the entry to senescence prevents the progression of aged cells that possess genetic damage by arresting their cell cycles [26]. However, due to defects in the normal clearance of the senescent cells mediated by the immune system, they eventually accumulate, and the combination of their paracrine and structural effects might result in the loss of function and carcinogenesis in the affected tissues [26].

Recent publications continue to support the rationale of the somatic mutation theory in which genetic alterations provide a fitness advantage to the affected cancerous cells. For example, mutations that increase expression of the genes involved in cell growth and proliferation, such as Myc, improve the proliferative rate of the cancerous cells, outcompeting the normal clones [27]. Modern DNA sequencing has identified over 4000 missense driver mutations in 240 genes in cancer genomes [28]; however, normal tissues have shown an unexpectedly large number of somatic mutations, which are usually found in neoplasms [29]. In addition, the current paradigm is challenged by other inconsistencies. For example, most oncogenes are essential players in the normal biology of cells [30], many carcinogens lack mutagenic effects [4], and cancer cells show morphological and transcriptional convergence regardless of the initial cellular phenotype [31], and the cancerous behavior can be acquired by events of transdifferentiation [32]. Moreover, is possible to revert to the malignant phenotype via the induction of differentiation using chemical agents [33], vitamins [34], transcription factors [35], or interactions with the extracellular matrix (ECM) and the stroma [36].

The limitations of the prevailing theory to explain the plasticity and convergence of the cancer cell have inspired novel proposals. For example, cancer is now increasingly conceived of as a disease initiated by genomic instability that allows the emergence of multipotent cancer stem cells [37,38,39] or envisioned as the pathological outcome of dysregulated pathways to justify the attainment of similar cancerous phenotypes [40].

Motivation for the hypothesis presented in this paper comes from the fact that many oncogenes induce senescence [41,42], and the process of EMT is sufficient to surpass it [43,44] and to acquire the cancer stem cell state from different phenotypes [19,45]. Furthermore, recent studies on carcinomas indicate that cellular senescence colocalizes with EMT [46], and, during the early process of carcinogenesis, cells co-express the biomarkers of both processes [47]. Therefore, in this contribution, the role of both processes is revisited to provide an alternative interpretation for the origin and progression of carcinomas in which the recovery of cellular plasticity is the main driver in the degeneration and carcinogenesis of the epithelial tissues.

Carcinomas arise from epithelial tissues and exhibit a robust pattern of cellular and molecular events [48]. In brief, the atypical hyperplasias are considered the precursors of the carcinomas in situ, which, in turn, may evolve from well-differentiated tumors into poorly differentiated and highly metastatic cancers [48,49,50]. Hyperplasias are characterized by the presence of senescent cells [51] that show beta-galactosidase (β-gal) and telomere attrition associated with replicative exhaustion [52] but are considered non-malignant tissues [53]. Their progression to carcinomas correlates with infiltration by cells of the immune system [54,55] and a diminution of senescent cells [56]. In turn, carcinomas tend to dedifferentiate and acquire aggressive behavior in a process accompanied by the gradual loss of epithelial features [48,49,50], while increasing the content of mesenchymal [57] and cancer stem cell biomarkers and the number of metastases [58]. Noticeably, the five-year survival rate in patients diagnosed with carcinomas exceeds the ninety percent, unless tumors present dedifferentiation, or they have already metastasized [59,60]. This is consistent with the fact that, in carcinomas, the increment of stromal cells [61] and EMT biomarkers [62] are independent factors of overall poor prognosis [63].

The current conception of senescence suggests that, rather than it being a mechanism that evolves to halt tumorigenesis, it is a type of cellular fate [16] in which cells acquire transient cellular plasticity [64], and, instead of being irreversible, cells eventually emerge from it [65]. Furthermore, the formation of sporadic carcinomas in vivo requires cellular senescence [66], and their pattern of hypomethylation has been considered primed for cancer behavior [67]. Interestingly, the abrogation of senescence has been linked with the overexpression of mesenchymal transcription factors, the induction of EMT, and the adoption of carcinogenic and invasive potential [43]. Moreover, the processes of senescence, inflammation, and EMT coexist in carcinomas [46], whereas presence of the biomarkers of stemness and inflammation has been involved in the acquisition of plasticity in senescent cells [68,69]. Recent analysis indicates that epithelial cells from the parenchyma undergo EMT and account for most of the fibroblastic cells in the desmoplasia associated with the carcinomas [70]. Likewise, in murine models, the majority of cancer-associated fibroblasts arise from the EMT of epithelial cells in vivo, and some acquire the genetic expression profile of cancer stem cells [71]. Additionally, the process of EMT has been reiteratively linked to the generation of mesenchymal stem cells and cancer stem cells [22,58,71], which, in turn, may give rise to fibroblasts, endothelial cells, pericytes, adipocytes, and macrophages in the tumoral microenvironment [22]. After experiencing EMT, cells also harbor the potential to migrate [72] and undergo mesenchymal-to-epithelial transition (MET) to produce metastasis with an epithelial phenotype upon exposition with the parenchyma of the lungs and bones [21,73]. Furthermore, cancer stem cells derived from EMT can reconstitute the entire tumoral mass along with its cellular heterogeneity [74]. Moreover, cancer stem cells are not only resistant to chemotherapy and radiotherapy but also induced after the exposition and may lead to secondary cancers or tumor relapse [75]. Hence, the modern perception of cancer considers the possibility that genomic instability, epigenetic reprogramming, or dedifferentiation allows the emergence of cancer stem cells that account for most of the features of the disease [37,38,39]. Likewise, the premise of the hypothesis is that the tumorigenesis and degeneration of epithelial tissues can be understood as the consequence of the discrete changes in cellular phenotypes fostered by the influence of cellular aging and inflammation on the machinery that controls the epigenetic profile. In essence, it is acknowledged that senescent cells are prone to dedifferentiation and that EMT epitomizes the process, enabling emergence of the mesenchymal cells involved in steatosis, calcification, fibrosis, and carcinogenesis of the tissues associated with aging.

Several lines of evidence suggest a conserved generic order of events involved in the recovery of plasticity in terminally differentiated epithelial cells; namely, their entry to senescence generates major epigenetic changes to then acquire the capabilities of the mesenchymal stem-like cells that potentially underlie the degeneration and carcinogenesis of epithelial tissues. Some of the strongest arguments that support the hypothesis are listed below. First, in human epithelial tumors as well as in cellular, organoid, *drosophila*, and murine models of carcinoma development, senescence precedes the malignant transformation of cells [65,66,76,77,78,79]. Oncogenes, radiation, chemicals, or high-fat diets induce cellular senescence from which cells then may acquire mesenchymal and malignant features [42,47,65,79,80]. Second, the process seems promoted by the immune system and involves activation of the molecular effectors of inflammation, epigenetic remodeling, and the process of EMT [54,81,82,83,84,85,86]. Third, during the early stages of carcinogenesis, senescent cells might co-express the mesenchymal or stem markers, and cancer-associated fibroblasts and cancer stem cells emerge by EMT [47,69,71]. Fourth, the mesenchymal cells arising from EMT can differentiate into adipocytes, osteocytes, and myofibroblasts or recover an epithelial phenotype and are highly resistant to current therapies [32,87,88].

The potential mechanistic connection between senescence, inflammation, and EMT during the genesis of the epithelial neoplasias has also been addressed by an in silico approach [89]. The regulatory dynamical model network indicates that gene regulatory constraints in the epithelial cells satisfy the attainment of three cellular and stable phenotypes. The time-ordered transitions correspond to the epithelial, senescent, and mesenchymal states. Interestingly, the computational analysis showed that the likelihood of converging into a mesenchymal stem-like phenotype increases when pro-inflammatory conditions are simulated. Hence, the study suggests that epithelial carcinogenesis emerges as a consequence of an underlying regulatory network and sheds light on the mechanism promoting the emergence of mesenchymal-like cells during inflammatory conditions [89].

This paper reviewed the molecular, cellular, and histological aspects of epithelial carcinogenesis to support the hypothesis that cellular senescence, inflammation, and the process of EMT are mechanistically linked with the emergence of cells that originate the carcinomas. In addition, it provides a plausible set of generic processes by which epithelial tissues degenerate via dedifferentiation and proposes a novel understanding of the phenomena of resistance by epigenetic switches that might spur further investigations and the development of new antineoplastic strategies.

## 2. Recovering the Plasticity of Epithelial Cells

Carcinomas arise from epithelial tissues and represent over 85% of all malignant tumors diagnosed in adults. The most common cancers develop from the skin, breast, endometrium, prostate, colon, lung, pancreas, bladder, liver, and cervix [90]. Broadly, epithelial tissues are constituted by the parenchyma, which are composed of epithelial cells and provide the specialized functions within an organ, and the mesenchymal cells which produce the scaffold by synthesis of the ECM [91]. A series of reports have indicated that the epithelium-specific transcription factors with a conserved ETS domain are crucial for the morphogenesis, development, and preservation of the epithelial phenotype [92,93]. Moreover, downregulation of the ETS specific to the epithelium (ESE) family of proteins is sufficient for the loss of differentiation and the generation of carcinomas [93]. In this regard, the transcription factors ESE-1 [94,95], ESE-2 [96], ESE-3 [97], and PDEF (prostate-derived Ets factor) [98] are reserved for epithelial cells, and their suppression induces the emergence of the mesenchymal phenotype [91]. Importantly, conditions that increase the expression of the zinc-finger binding transcription factor (Snail), the Snail family transcriptional repressor 2 (Slug), the Twist family bHLH transcription factor 1 (Twist1), the Twist-related protein 2 (Twist2), the zinc finger E-box-binding homeobox protein 1 (ZEB1), and ZEB2 promote downregulation of the ESE proteins and the emergence of fibroblasts [99]. The process by which epithelial cells undergo transdifferentiation into mesenchymal cells is called EMT, and it is part of the normal process of gastrulation in the early embryo development. Of note, mesenchymal cells migrate through the embryo for proper morphogenesis, and some undergo MET to originate the endoderm, while others remain differentiated in the mesenchyme to generate the mesoderm [100]. Hence, under certain conditions, normal epithelial cells dedifferentiate and transdifferentiate in similitude to cells derived from carcinomas [101,102].

The hypothesis claims that cellular aging allows the recovery of cellular plasticity on terminally differentiated epithelial cells. This assumption considers that epithelial tissues require a high proliferation rate to sustain their functions. Hence, they eventually accumulate senescent cells and generate hyperplasias [103]. These lesions are considered the non-obligated precursors of the carcinomas [104] and are characterized by the presence of short telomeres and other features associated with cellular senescence [51,105]. In addition, senescent cells and the levels of p16 progressively accumulate with age [106] and correlate with a decline in the replicative capacity of epithelial tissues [107]. Furthermore, the relationship between aging and cancer is highlighted by the incidence curves for most carcinomas since they rise after the age of 50 years, and chances increase exponentially with each extra decade of life [108]. Hence, the molecular process that cells undergo during senescence in hyperplasia is mechanistically linked to the fundamental causes of carcinoma development in vivo [76].

The molecular pathways activated in response to replicative exhaustion are similar to a DNA damage response (DDR) [109]. The erosion of telomeres initiates a series of actions that would bring cell cycle arrest [109]. As such, senescent cells display structures in the telomeres, known as DNA repair foci, that are also observed in response to DNA double-strand breaks [110]. Their generation involves the phosphorylation of the histone family member X (H2AX) by the Ataxia telangiectasia mutated protein kinase (ATM) [111], the activation of p53, the checkpoint kinase 2 (Chk2) [112], the cyclin-dependent kinase inhibitor 1 (p21) [113], the promyelocytic leukemia protein (PML) [114] and the epigenetic derepression of the locus of the inhibitors of kinase (Ink4b/ARF/Ink4a). The locus encodes the cyclin-dependent kinase inhibitor 2B (p15), the cyclin-dependent kinase inhibitor 2A (p16), and the alternative reading frame product of CDKN2A (p14) [106]. Intriguingly, the activation of ATM also triggers the transcription nuclear factor kappa B (NF-κB) by the phosphorylation of the NF-κB essential modulator (NEMO) [115]. In turn, this protein coordinates the secretion of cytokines, chemokines, growth factors, and proteases liberated by senescent cells [15,116]. In addition, senescent cells experience changes in the configuration of chromatin that are associated with genome instability [117] and that might also be influenced by NF-κB since it binds the promoters of demethylases and conducts transcriptional derepression [118]. For example, the histone H3 lysine-27 demethylase Jmjd3 (Jmjd3) depends on the direct binding of NF-κB to a cluster of three kB sites in its promoter and, upon activation, promotes histone demethylation, which leads to cellular plasticity [118]. Histones participate in the regulation of gene expression, and their post-translational changes influence the chromatin configuration and the genetic transcription [119]. Additionally, the structural and numerical centrosome abnormalities in senescent cells also contribute to the secretion of multiple inflammatory factors and genomic instability [120].

Inflammation drives cellular senescence, and, thereafter, cells may adopt the morphology of a fibroblast with the upregulation of mesenchymal and stem biomarkers [84]. Further, overexpression of the mesenchymal transcription factors is reiteratively linked with the suppression of cellular senescence through EMT and the acquisition of tumorigenic and invasive potential [43]. Notably, the promoters of the mesenchymal transcription factors also contain binding sites for NF-κB [120], and the constant stimulation of Snail, Slug, Twist, or Zeb originate repression of the Ink4b/ARF/Ink4a locus and ETS proteins [121,122,123]. Moreover, the mesenchymal transcription factors bind and repress the promoters of the ESE proteins [124], whereas the suppression of the cellular senescence seems to be related to activities of the polycomb proteins that modulate the structure of chromatin [125]. Remarkably, the axis of NF-κB, Twist, and the proto-oncogene polycomb ring finger protein 1 (Bmi1) resulted as being essential for the repression of the epithelial phenotype, the Ink4b/ARF/Ink4a locus, and to sustain the transcriptional signature associated with a mesenchymal stem cell [85]. Interestingly, Bmi1 regulates stemness in breast cancer cells by the positive modulation of the homeobox pluripotency transcription factor (Nanog) expression through the NF-κB pathway [126].

Perhaps, the strongest evidence supporting the ability of aged cells to recover plasticity after cellular senescence by endogenous processes came from the studies in primary cultures of normal epithelial cells. The phenomenon of spontaneous immortalization implies a discrete change in phenotype, which epithelial cells experience in vitro after replicative exhaustion [70]. During this process, the gradual loss of epithelial markers in response to senescence is observed, from which the cells eventually arise with a fibroblastic morphology expressing the mesenchymal biomarkers [127]. Current findings show that proinflammatory cytokines induce proliferation on epithelial cells and eventually on their entry into cellular senescence [84]. Increasing the interleukin concentration drives direct EMT in cells with the adoption of the fibroblastic morphology and functional properties of migration, invasion, and stemness [84]. Moreover, in cells from carcinomas, the response to genetic damage induces inflammation and a mesenchymal stem malignant phenotype [115].

**Figure 1:**
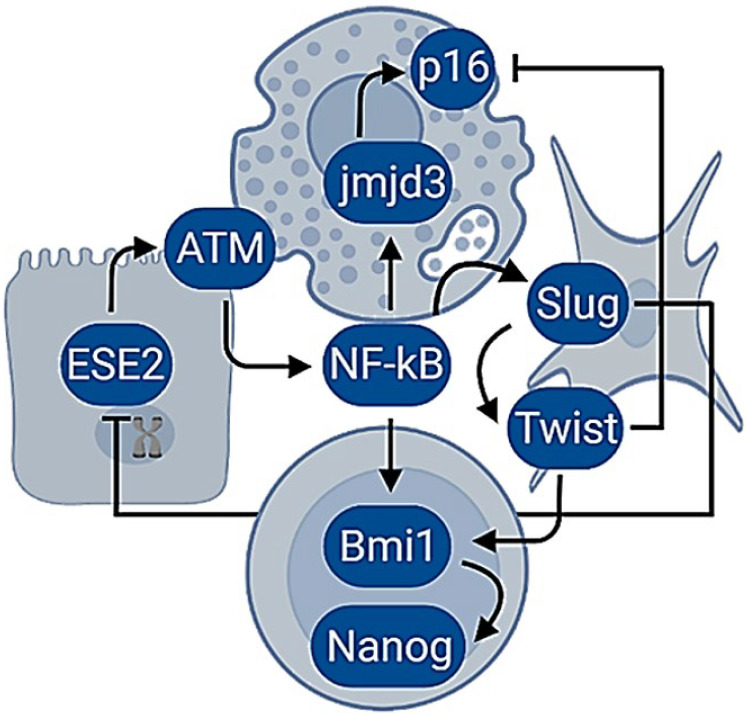
Molecular interactions potentially involved in the recovery of cellular plasticity in aged epithelial cells. According to the hypothesis, the constitutive activation of the DDR and NF-κB enables aged cells to undergo epigenetic switches into mesenchymal and stem phenotypes. In addition, in response to oscillations in endogenous or microenvironmental stimulus, the aged epithelial cells might give rise to several phenotypes and lead to tumoral heterogeneity and metastasis.

The hypothesis suggests that senescent cells dedifferentiate when the DDR response and NF-κB are constitutively activated. Hence, it proposes to link aging and inflammation with carcinogenesis by the activation of an endogenous molecular network in which differentiated phenotypes can manage the recovery of cellular plasticity (Figure 1).

In line with those suppositions, the adoption of mesenchymal stem traits and malignancy in epithelial cells seem to require the presence of genetic damage [115] and inflammation [84]. In addition, the NF-κB signaling pathway in carcinoma cell lines produces demethylation and upregulation of the genes normally expressed in the pluripotent stem cells [128]. In the same way, the stimulation of IL-6 drives epigenetic changes dependent on NF-κB and DNA methyltransferases that resulted in epigenetic reprogramming and the emergence of cancer stem cells [129]. Furthermore, the activation of NF-κB by IL-6 was sufficient to induce an epigenetic switch from breast immortalized cells to cancer stem cells in a single event of dedifferentiation [130].

The transcription factor NF-κB is well known for its critical role in mediating responses to a remarkable diversity of stresses [131]; however, is has emerged lately as the central hub in the molecular network that coordinates the events of transdifferentiation induced by inflammatory cytokines and the generation of cancer stem cells [129].

In stem cells, plasticity is associated with transcriptional hyperactivity mediated by the combined effects of the polycomb proteins, DNA methyltransferases, and transcription factors on the configuration of the chromatin [132,133,134]. In addition, the emergence of stemness and its maintenance is strongly influenced by several unspecific stressors, such as the level of cytokines, oxygen, growth factors, mechanical forces, DNA damage, radiation, and antineoplastic agents [135,136,137,138,139]. Therefore, stemness can be viewed as a cell state rather than a fixed phenotype [132]. Furthermore, the normal biology of the stem cells has been implicated in most features associated with cancer. For example, their metabolism relies on glucose and glutamine to proliferate and is characterized by the conversion of glucose to lactate, despite the presence of enough oxygen to sustain its complete oxidation [140,141]. In stem cells, the proteins p16 and p53 are downregulated [142,143], while the overexpressing process of DNA repair, detoxifying enzymes, and ABC transporters results in an increased threshold for apoptosis [144]. Additionally, telomerase is reactivated and implicated in cellular immortality [145]. Moreover, throughout long-term cultures, stem cells develop chromosomal instability [146] and possess the constitutively high levels of NF-κB necessary to sustain the undifferentiated phenotype [147]. Finally, stem cells self-renew or differentiate into several cellular phenotypes according to microenvironment cues [147]; they survive the anoikis and exhibit an inherent ability to migrate [148], along with intrinsic capabilities to evade the immune response [149].

Senescent cells exhibit major changes in the chromatin configuration mediated by the effects of the polycomb proteins [150] and an increment in the transcriptional capabilities associated with demethylation and other epigenetic mechanisms that might underlie their susceptibility to malignant transformation [67]. Altogether, it is argued that senescent cells resemble some aspects of the stem state; the hypothesis suggests that in response to molecular damage, inflammation triggers an epigenetic reprograming in aged epithelial cells that enables the recovery of cellular plasticity and the behavior of cancer stem cells. On the other hand, at the histological level, cellular senescence disrupts the structure and endocrine environment. In the following, the evidence regarding the tendency of epithelial tissues to degenerate in the presence of senescent cells is reviewed, and the hypothesis provides a novel explanation for a potential series of events that might shape the carcinogenesis of the epithelial tissues in vivo.

## 3. The Progression of Epithelial Hyperplasias into Carcinomas

Senescent cells might adversely influence the differentiation and behavior of neighboring cells by disrupting the endocrine and structural microenvironment, which, along with their recovered cellular plasticity, may foster malignant transformation of the epithelial tissues affected by hyperplasias. In addition to the process repeatedly associated with the malignant progression of the hyperplasias is the infiltration of lymphocytes [81,151,152]. The attraction of immune cells to tissues bearing senescent cells is comprehensible since their secretions include the monocyte chemoattractant protein 1 (MCP-1) and matrix metalloproteinases (MMPs) that stimulate the migration and infiltration of monocytes, lymphocytes, and NK cells, along with the degradation of the ECM, proteinase inhibitors, cell surface receptors, and cell–cell adhesion molecules [153]. In turn, activation of the macrophages may drive an increased expression of proinflammatory cytokines and MMPs [154], both of which are associated with the induction of EMT [18,19] and the generation of cancer stem cells [19,155]. The cytokines’ tumor necrosis factor-alpha (TNF-α), the transforming growth factor-beta (TGF-β), and the interleukin-6 (IL-6) are some of the most studied inducers of EMT [83,156,157,158]; all of these converge in the activation of the transcription factor NF-κB [156,157,159]. Regarding MMPs, their effects on the induction of EMT also require the activation of the mesenchymal transcription factors by NF-κB [160]. Another event that favors dedifferentiation via EMT is cell detachment from the ECM, the basal membrane, or other cells [18]. The mechanical stress imposed in those conditions generates cancer stem cells through activation of the focal adhesion kinase (FAK), the transcription factor Yes-associated protein (YAP), and the transcriptional co-activator with PDZ-binding motif (TAZ) [161]. Another mechanism potentially involved in the progression of hyperplasias is the coupling of the integrin and Rac kinases with the NF-κB pathway through the IKK complex [162].

The processes of senescence and EMT are associated with increased activity of NF-κB [83,163] and linked to events of the early development or the recovery of cellular plasticity [64,164,165]. In line with those observations the hyperactivation of the NF-κB pathway in tumors is comprehensible since this protein responds to structure or endocrine changes in the environment, including damage in the stroma, the levels of cytokines, radiation, oxidative stress, hormones, and growth factors, or due to intracellular molecular damage [166].

Here, it is hypothesized that the structural disruption in tissues with the presence of senescent cells may bring constant inflammation to facilitate events of dedifferentiation that manifest as mesenchymal cells that might lead to cancer. In a sense, in the beginning, senescent cells are primed for cancer behavior due to endogenous damage. However, eventually, their presence in tissues produces an aberrant microenvironment that may promote and amplify the dedifferentiation of non-aged cells via extracellular signals but essentially through the same molecular interactions (summarized in Figure 1).

Cells from carcinomas adopt the mesenchymal-stem phenotype in response to DDR, ATM, and NF-κB activation and produce IL-6, which promotes persistent proliferative signaling and the fibroblastic shape [115]. Intriguingly, fibroblasts detected in preneoplasic lesions have been shown to express the markers of senescence and the alpha-smooth muscle actin (α-SMA) [47]. This last protein is associated with myofibroblasts, which are mesenchymal cells that harbor enhanced plasticity and multipotency [70,167,168]. In the case of high-grade carcinomas, the loss of epithelial features and the increment of the mesenchymal and cancer stem cell biomarkers [58,62,63] are typical. In addition, the gain in the stromal compartment is an independent factor of overall poor prognosis and the presence of metastasis [61,62,63].

Fibroblasts in the tumoral microenvironment are traditionally viewed as cells promoting inflammation, EMT, metastasis, angiogenesis, and the enrichment of cancer stem cells [169]. Given that fibroblasts in carcinomas are mostly derived from epithelial cells that undergo EMT [71], and that the increment of pro-inflammatory cytokines correlates with EMT and the accumulation of cancer stem cells within tumors [19], the hypothesis provides an alternative interpretation for the origin of the stromal cells associated with carcinomas in which the combination of endogenous molecular damage and structural tissue disruption in aged epithelial cells promotes higher levels of constitutive inflammation that favors the attainment of a mesenchymal–plastic phenotype. Therefore, it also may explain the epithelial dedifferentiation and the stromal enrichment observed during the histological progression of carcinomas.

Cancer-associated fibroblasts are characterized by increased NF-κB expression and the secretion of TGF-β, IL-6, MCP-1, and MMPs [71,169,170], whereas mesenchymal cells arising from EMT tumors in vivo showed higher degrees of plasticity and multipotencyand lower DNA methylation; some may acquire the genetic expression profile related to the cancer stem cells [19]. The mesenchymal cells produced by EMT express the pluripotent genes SRY-box transcription factor 2 (Sox2), Nanog, the octamer-binding transcription factor 4 (Oct4), and the neurogenic locus notch homolog protein 1 (Notch1) [32,171]. Noticeably, the cellular plasticity endowed by inflammation not only activates the EMT of epithelial cells, but also, upon enough stimulation, the endothelial cells, monocytes, fibrocytes, pericytes, adipocytes, and local fibroblasts can dedifferentiate into myofibroblasts [70,172]. In turn, the adoption of the fibroblastic program confers cells with the intrinsic potential to then differentiate into several subtypes of mesenchymal and non-mesenchymal lineages [173,174].

Normal fibroblasts display transcriptional diversity and heterogeneity relative to the mechanical forces they experience due to structural changes, position, and the endocrine milieu in different tissues [173], and, under appropriate conditions, they can differentiate into adipocytes, chondrocytes, osteocytes, hepatocytes, neurons, myocytes, or pancreacytes [175,176]. Cells in the mesenchymal program possess the ability to migrate and infiltrate into damaged tissues, in which they may differentiate into fully functional cells [177,178] but may also generate tumors [179,180]. Likewise, the formation of metastasis is understood as cells from tumors that undergo EMT and then migrate until reversion of the process in response to the microenvironmental changes by MET that re-enables the adoption of the epithelial phenotype [21,181]. In this regard, during the reversion of EMT, cells interact with the parenchyma, which downregulates the expression of the mesenchymal transcription factors, thereby allowing recovery of the epithelial phenotype [20,73].

Some cells derived from carcinomas require higher levels of proinflammatory cytokines to sustain the fibroblastic shape, malignancy, and stemness [115]. Accordingly, the suspension or diminution of inflammation resulted in the reappearance of the epithelioid morphology [84,182]. Further, the activities of Twist1 and Slug seem necessary for the overexpression of Bmi1 and to retain the stemness and invasiveness. In the same way, the inhibition of the mesenchymal transcription factors results in the emergence of the luminal (epithelial) phenotype [183]. Altogether, the endogenous tendency of the most malignant subtypes of carcinomas to adopt a basal (fibroblastic) shape derives from DNA damage that, in turn, generates a permanent ATM activation and constitutive higher levels of NF-κB, secretion of cytokines, and the acquisition of the features of a mesenchymal stem cell with malignant behavior [115]. Noteworthily, their invasive potential and disorganized growth have been associated with genes that require the activity of NF-κB [184]. Intriguingly, the combined effect of an artificial ECM and the chemical interference of the inhibitor of nuclear factor-κB (IκB) kinase (IKK) complex was able to restore the epithelial phenotype, the formation of organized clusters, and suppression of the malignancy and motility [184].

The potential relevance of tissue and stromal architecture over cell behavior [185], as well as the role of cell dedifferentiation into myofibroblasts for the origin of cancer, has been previously explored [186]. Interestingly, both hypotheses postulate that mutations are unnecessary for cancer development and arise as byproducts of the process of carcinogenesis [185,186]. Nowadays, the major role of the cell microenvironment and inflammation in cellular plasticity is increasingly accepted [162,184]. Furthermore, the molecular regulatory networks that coordinate the events of transdifferentiation and the generation of cancer stem cells have been continuously elucidated. Expectedly, they show convergence into the activity of NF-κB and its influence on the regulators of the epigenetic control [83,115,129,163]. In addition, stemness remains elusive to describe in vivo since requires special conditions [187]. For example, its emergence and maintenance are strongly influenced by the dynamics of cell growth, which, in turn, naturally induces cellular heterogeneity due to cell–cell interactions and the oscillatory level of cytokines, oxygen, growth factors, and mechanical forces [188,189,190]. On this subject, the concept of niches captures the notion of stem cells as entities that are highly dependent on microenvironmental conditions, since it describes domains in which cells and their structural and endocrine interactions result in stemness [187]. The fact that the maintenance of stem cells in vitro requires serum-free media enriched with growth factors, such as TGF-β, TNF-α, and hypoxic conditions; to avoid their differentiation, illustrates the challenge that exists in defining their nature [191]. Moreover, the comparison of cancer stem cells and embryonic stem cells revealed convergence at the transcriptional [192] and functional levels [193]. Therefore, it has been suggested that the only difference between the normal and the cancer stemnesses lies in the tumor microenvironment [194]. Recent evidence shows the existence of hybrid phenotypes within carcinomas that possess the ability to regenerate tumors with the original cellular heterogeneity [195]. This finding is expected considering the molecular biology of stem cells, since their transcriptional hyperactivity results in the expression of markers from several lineages [134].

**Figure 2:**
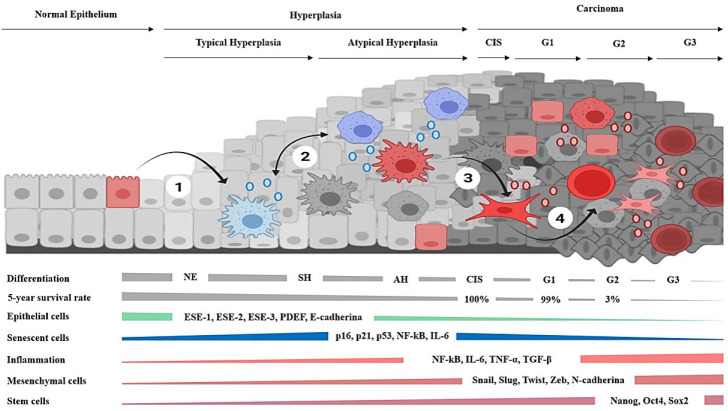
Molecular and cellular processes that may shape the histological progression of carcinomas. The hypothesis considers that epithelial tissues exposed to proliferation eventually become enriched with senescent cells and conduct into the hyperplasia (1). In this setting, cytokines generate infiltration of the immune cells (2) that potentially induce EMT in senescent cells (3). After the EMT produced by inflammation cells acquire the fibroblastic program and cellular plasticity that is evident with the emergence of stem biomarkers (4). The influence and fluctuations of several stimuli due to changes in the tumoral microenvironment may give rise to cellular heterogeneity. For example, the interactions with stroma, cells, parenchyma, and the endocrine milieu, including the concentration of oxygen, nutrients, and growth factors or the reduction in the levels of inflammation, would originate the emergence of epithelial, fibroblastic, myofibroblastic, hybrid or stem phenotypes. However, in the context of the high-grade carcinoma, the undifferentiated tumorigenic state is preferentially sustained by the burden of endogenous molecular damage and an increased inflammatory microenvironment that includes tissue disruption. Hence, the apocrine and basal tumors lose the glandular architecture because the mesenchymal and pluripotent transcription factors are constitutively activated, thus explaining the increment in stromal content along with the metastatic and aggressive behavior. Bars illustrate the degree of differentiation and their relationship with survival as well as the molecular biomarkers that characterize each cellular process with their relative abundance during every histological stage in the progression of carcinomas. NE, normal epithelia; SH, simple hyperplasia; AH, atypical hyperplasia; CIS, carcinoma in situ; G, grade.

The hypothesis proposes that epithelial carcinogenesis, at its core, is fostered by gradual transcriptional derepression in aged or damaged cells, which, in conjunction with the microenvironmental cues, drives and shapes the histological progression of carcinomas. The setting of the high-grade tumors favors the existence of deregulated plasticity and access to stemness. Therefore, the advanced phases of neoplasias that arise from the epithelial tissues adopt an apocrine and basal phenotype (Figure 2).

The events underlying the entire histological progression of carcinomas in vivo remain partially understood. Here, a plausible molecular mechanism by which aged cells recover cellular plasticity due to constant activation of DNA damage response and inflammation is described. In addition, the hypothesis considers that, in conjunction with the immune system and stromal disruption, senescent cells promote tissue dedifferentiation and explain the progression of hyperplasias into carcinomas. Instead of considering that the cancerous phenotype is the result of mutations or selective gene modulation, it is proposed that the replicative cellular senescence leads to transcriptional derepression, which leads to plasticity and discrete changes in cellular phenotypes.

The hypothesis describes a plausible molecular mechanism by which aged cells recover cellular plasticity due to constant activation of the DNA damage response and inflammation. Both processes will promote dedifferentiation and the progression of hyperplasias into sporadic carcinomas. Hence, the cancerous phenotype may result from transcriptional derepression mainly due to replicative cellular senescence. The mechanism involves the linking of inflammation with the DDR response and epigenetic remodeling. Hence, this process will lead to plasticity and discrete changes in cellular phenotypes. The emergence of either myofibroblast, basal, or stem phenotypes from aged cells might be responsible for the cancerous behavior, rather than attributing to mutations the creation of malignant traits to mutations. The hypothesis explains the long relationship of cancer with genetic damage, aging, and inflammation via the connection of chromosomal damage with the secretory phenotype and the immune system’s infiltration in response to it. Under the hypothesis, the increased cancer incidence with age, the long latency period, and the multistep process of carcinogenesis result from replicative exhaustion that leads to hyperplasias. In addition, it considers that the progression into invasive carcinoma requires higher levels of inflammation to enable increased chromatin derepression and cellular plasticity. These conditions may give rise to the histological atypia and infiltration associated with the malignant transformation of hyperplasias. Early in the process of carcinogenesis, a premise is that malignant behavior arises from senescent cells undergoing EMT in hyperplasia. However, the dedifferentiation of several phenotypes is expected in the conditions associated with high-grade carcinomas. Hence, nearby or migrating cells may contribute to tumors in the absence of telomere attrition. The hypothesis suggests that, in the setting of the apocrine or large-sized tumors, the effects of inflammation and tissue disruption during the differentiation state of cells account for the aggressive and self-sustained malignant behavior of the advanced carcinomas. Permanent activation of these conditions would facilitate the gradual loss of epithelial differentiation. Hence, carcinoma progression correlates with increased stromal, inflammatory, EMT, and stem cell markers. According to the molecular mechanisms reviewed, under a certain threshold of endogenous molecular damage and tissue disruption, epithelial tumors evolve into the autonomous entities that characterize invasive and metastatic deadly carcinomas.

## 4. Demystifying Tumor Heterogeneity and Resistance

Broadly, the advanced phases of carcinomas remain untreatable, and the management of stage I to III tumors results in their eventual progression with the development of the incurable metastatic disease [196]. In addition, most antineoplastic drugs and radiotherapy are clastogenic and carcinogenic [7,197], and the deterioration post-therapy has been estimated during 15 years of cellular aging [198]. Hence, those who survive cytotoxic therapies may experience the emergence of secondary cancers or tumor relapse, extensive organ fibrosis, accelerated aging, and early onset of its associated diseases [7,197,199,200,201]. Moreover, substantial evidence suggests that therapy is an active player in the changes that undergo the malignant and premalignant cells. In this regard, chemotherapy and radiotherapy not only generate the DNA damage that results in tumoral shrinkage and cell death associated with the partial or complete responses, but they also simultaneously trigger senescence, inflammation, EMT, and stemness in the exposed cells, which manifest with the adoption of different epigenetic profiles and increased aggressiveness [23,46,136,202,203].

Recent insights from breast carcinomas are consistent with the notion of epigenetic switches, rather than mutations and selection as the driving force of malignancy and resistance [204]. Furthermore, it has been suggested that instead of targeting multiple signaling pathways associated with resistance in the heterogenous tumoral microenvironment, a better approach would be to prevent the entry of cancer cells into the cellular states that allow them to survive therapeutics [205]. This section brings evidence from breast neoplasia that links cellular aging and inflammation not only with the genesis and progression of carcinomas but also with their heterogeneity, resistance, and relapses after therapy. In addition, considering the insights derived from the hypothesis and the current state of knowledge in breast epithelial biology and its neoplasms, an epigenetic landscape is proposed that gives rise to the molecular heterogeneity and progression in breast carcinomas despite the application of therapy.

According to morphological features, breast carcinomas exceed the 20 subtypes; however, over 90% are histologically classified as invasive ductal carcinoma or lobular carcinoma. The rest are considered rare, such as the medullary, metaplastic, apocrine, mucinous, cribriform, tubular, neuroendocrine, and pleomorphic [206]. Regarding the histological grade, the assessment of the degree of differentiation is used to stratify breast cancers into grade 1, for slow-growing and well-differentiated tumors; grade 2, for moderately differentiated; and grade 3, for highly proliferative and poorly differentiated [207]. Based on gene expression profiles, four molecular subgroups are well established, named Luminal A, Luminal B, HER2+, and triple-negative breast carcinomas (TNBC) [208]. In general, Luminal A and B comprise over 70% of newly diagnosed breast cancer cases, express hormonal receptors, and have the characteristics of luminal epithelial cells of the breast. The HER2+ variant represents 20% of newly diagnosed breast cancer cases and is characterized by a high expression of the protein HER2 and a loss of the hormonal receptors in tumors of greater histological grade [209], but they also can be classified as luminal type [210]. In the case of the TNBC, they account for approximately 10% of all cases and lack the expression of the hormonal and HER2 receptors; the luminal differentiation markers are claudin-low, and TNBC is considered the most aggressive subtype of breast carcinoma [211]. These tumors display, from the beginning, strong resistance to chemotherapy and radiotherapy [212]; histologically, they show high-grade, basal markers, fibrotic zones that push the borders of invasion, and lymphocytic infiltration [213] with vimentin and NF-κB expression [214]. Considering the gene expression profile of TNBC, they display signatures that correspond to myoepithelial markers and DNA damage responses, overexpression of immune signal transduction pathways, EMT and mesenchymal stem genes, and a subgroup enriched in hormonal pathways [215].

The differences in the molecular subtypes of breast carcinomas have been exploited to assess prognosis as well as for a strategy for therapy selection; however, the responses are temporal and followed by relapse. Interestingly, the acquisition of resistance and malignancy is accompanied by changes in the original molecular profile [88,216,217]. Moreover, TNBC tumoral masses contain cells that correspond with the other subtypes, with an estimated composition of 50% basal-like, 30% claudin-low (both considered TNBC), 9% HER2+, 6% luminal B, 5% luminal A, and remnants of normal-like tissue [215]. Furthermore, cumulative evidence demonstrates the interconversion among breast cancer subtypes and is illustrated by the emergence of resistance. For example, the luminal subtypes become insensitive to the antiestrogen tamoxifen by EMT and the adoption of the epigenetic profile of the mesenchymal stem cell [216]. Similarly, breast cancer patients whose primary tumor was HER-2 negative turn positive during cancer progression [218]. Likewise, HER2+ breast cancers evolve into TBCA tumors during the treatment with trastuzumab, the humanized antibody directed against the extracellular domain of the tyrosine kinase receptor HER2. In this case, the process of resistance also involves EMT with and independence from the ERBB family signaling pathway. Hence, drugs induce EMT and the emergence of the TNBC phenotype along with increased resistance, stem cell features, and metastatic potential [88]. On this matter, analysis of the primary breast cancer biopsies, including triple-negative specimens, revealed the enrichment of cancer stem cell signatures after chemotherapy [219]. Notably, the molecular responses associated with the resistance of TNBC found an increment in the expression of β-gal, p16 [220], and the proportion of polyploid senescent cells in tumors that failed to respond to neoadjuvant chemotherapy [221]. Moreover, in a pre-clinical model of TNBC, a causal relationship was demonstrated between therapy-induced senescence and the generation of chemoresistant stem-like populations [222]. The exposition of TNBC cells to doxorubicin also induces the EMT transcription factors and increases their resistance to treatments [223]. Tumors conformed mostly by triple-negative cells are the most lethal subtype of breast cancer and lack effective therapeutic options [212]. Chemotherapy, radiotherapy, and targeted drugs fail to improve prognosis; and the progression-free survival remains low with a median of 7 months [224]. TNBC tumors poorly respond to tamoxifen, trastuzumab, or Atezolizumab, a humanized monoclonal antibody against the programmed death-ligand 1 (PD-L1), which negatively regulates the cytotoxic T-lymphocyte activation [224].

Understanding the origin of carcinomas has the potential to postulate novel approaches to prevent and treat neoplasia. The hypothesis and the reviewed evidence suggest that carcinomas arise from a few preexistent and endogenous cellular states. In addition, studies indicate that senescence and EMT enable not only the progression from hyperplasia to neoplasia but also the evolution of carcinomas by dedifferentiation into higher grades in which the stem or basal subtypes are naturally resistant to current therapeutics.

Providing further support for those statements, in a recent transcriptomic characterization of the normal breast tissue, it was proposed that the basal mammary stem cells differentiate either into myoepithelial cells or luminal progenitors, which gives rise to the two distinct luminal cell types, one secretory and the other hormone-responsive [225]. Moreover, they found correspondence between the discovered normal cellular states with the molecular subtypes of breast carcinomas [225]. Given that the exposure of immortalized breast epithelial cells to IL-6 is sufficient to induce an epigenetic switch mediated by NF-κB and the emergence of TNBC-like cells [130], it appears that any attempt to treat or prevent cancer that produces toxicity has the potential to result in the dedifferentiation of normal and cancerous cells with the adoption of preexisting and robust malignant cellular states.

The attainment of the stem and basal phenotypes involves persistent activation of the DNA damage response [115], which is the disruption of tissue resulting in constitutively higher levels of inflammation that lead to stemness [115,162]. In accordance, TNBC tumors exhibit an enriched expression of DDR, inflammation, EMT, and stemness [215]. In addition, the acquisition of the fibroblastic shape in cells derived from carcinomas seems to require inflammation [84,182], the DNA damage response, and the activity of ATM, NF-κB [115], and the EMT transcription factors [183]. Consistently, most TNBC cell lines are assigned as basal or mesenchymal-like subtypes and display the expression of EMT and stem markers [226].

**Figure 3:**
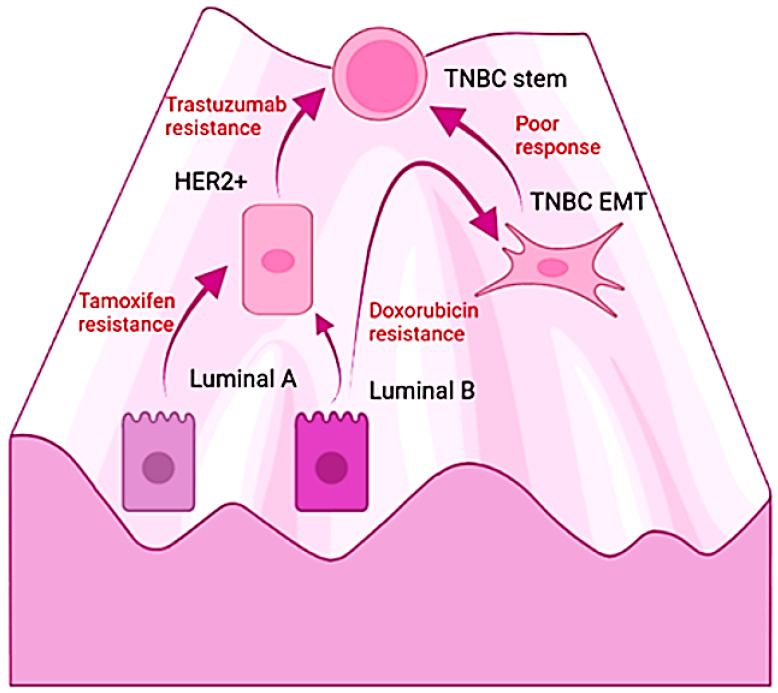
The potential landscape for breast cancer progression and epigenetic switches involved in the emergence of resistance. The recent characterization of the transcriptomic profiles in the normal breast suggests the existence of five cellular states derived from a basal stem cell that differentiates into a myoepithelial cell or a luminal progenitor, from which two types of luminal cells arise. The information from this pathway of differentiation was used to illustrate the cellular heterogeneity and their relative hierarchy within the normal breast. Then, breast cancer subtypes were located according to their molecular profile in the basin of the normal transcriptional-related counterpart to highlight that complexity and the heterogeneity of breast carcinogenesis may be understood as the reversion of differentiation. Finally, some of the reviewed events of dedifferentiation in response to cancer therapy are depicted in purple arrows. Remarkably, the processes of cellular senescence and EMT are also involved in triggering the epigenetic switches among the molecular subtypes of breast cancers.

Considering those recent findings, the hypothesis suggests that we can understand the malignant transformation of the epithelium and the progression of carcinomas as the permissive access of aged cells into endogenous cellular states within the boundaries of the pathway of differentiation depending on the type of stimulus. It speculates that cellular senescence and EMT allow those epigenetic switches and explain the evolution of luminal or HER2 subtypes of breast cancer into the lethal TNBC tumors (Figure 3). Furthermore, it explains the ability of IL-6 to recover the TNBC phenotype from the non-cancerous cells derived from hyperplasias and primary cultures. Importantly, the coupling of damage with NF-κB, epigenetic regulators, and the mesenchymal transcription factors provides a molecular mechanism by which to explore novel approaches for the management of carcinomas. In other words, the hypothesis suggests that targeting the degree of malignancy with drugs that modulate inflammation may restore the epithelial differentiation of high-grade tumors along with a diminution in their malignancy in spite of the endogenous molecular damage and the aberrant extracellular signals in the tumoral microenvironment.

On this subject, leukemias and hematological disorders are naturally understood as diseases of immature lymphoid or myeloid cells; hence, the implementation of differentiation therapy seems reasonable [227]. Conversely, solid tumors are neither considered cancers generated by dedifferentiated precursor cells nor able to differentiate due to their aberrant genetic code [228]. However, according to the reviewed mechanisms, slowing cancer progression and improving survival seem probable if therapy focuses on downmodulation of the inflammation, since this will result in partial recovery of the histological differentiation and the prevention of aggressive behavior.

Consistent with that view, the combined effect of an artificial ECM and the pharmacological inhibition of NF-κB restores the epithelial phenotype and the organized growth while preventing the malignant phenotype of breast cancer cells [184]. Accordingly, tissue architecture exerts a major influence over the genotype and behavior as demonstrated by the effect of the ECM on restoring a normal phenotype in mammary epithelial cells [229]. Similarly, in a murine model of hepatocellular carcinoma, the inhibition of LIF/JAK1/STAT3 and NF-κB signaling pathways induced the differentiation of cancer stem cells and inhibited their self-renewal and tumorigenic capacity [230]. Moreover, the modulation of the NF-κB signaling and the suppression of the activities of the polycomb protein EZH2 resulted in enhanced differentiation of nasopharyngeal carcinoma cells in vitro via epigenetic mechanisms [231]. Epithelial tumors can regress by differentiation after their implantation in normal tissues [232]. The hypothesis argues that the combined effects of the ECM and epithelial cells over the activity of NF-κB result in its downregulation and the partial recovery of differentiation.

In summary, from the atypical hyperplasia to the higher-grade carcinomas, the inflammation associated with the genetic damage of aged cells appears to drive the carcinogenesis in the epithelial tissues by triggering cellular plasticity and dynamical events of dedifferentiation and transdifferentiation, which may underlie tumoral heterogeneity, progression, and resistance. Despite breast carcinomas having been widely considered from different molecular and cellular origins or promoted by several genetic alterations, tumors intrinsically possess the other variants that, in turn, show correspondence with few preexistent cellular states in the normal breast tissue. Furthermore, during progression, or in response to cancer therapy, it seems that breast cancer cells undergo epigenetic switches from one cellular state into another according to the stimulus. Therefore, cellular senescence, inflammation, and EMT might be involved not only in the malignant transformation of the epithelial hyperplasias into carcinomas in situ, but also, in the acquisition of an increasing degree of dedifferentiation during the progression of carcinomas. Considering the reviewed evidence, it is plausible that TNBC entities corresponded not only with cancers originating from the basal mammary stem cells or the myoepithelial cells but also that they reflect the behavior of breast cells in the conditions of molecular and structural damage. Hence, adversity may prompt the emergence of the robust mesenchymal or basal phenotype primed for the lethal cancer behavior that is resistant to current therapeutics. In the light of the mechanistic role proposed in the hypothesis for cellular aging and inflammation during carcinogenesis, it seems plausible that the failure of antineoplastic therapy results from the link between stress and molecular damage with transcriptional derepression and stemness. Hence, cytotoxic drugs or radiotherapy may foster an epigenetic switch in the surviving cells into a more resilient cellular phenotype. Additionally, the emergence of aggressive tumor relapses is comprehensible, since the clastogenic events derived from them stimulate higher levels of inflammation and plasticity. Interestingly, the adoption of mesenchymal lineages that substitute the epithelial phenotypes suggests a generic principle for cell behavior in terminally differentiated cells once they have experienced replicative cellular senescence or non-lethal adversity.

## 5. A Misunderstood Process of Development

**Figure 4:**
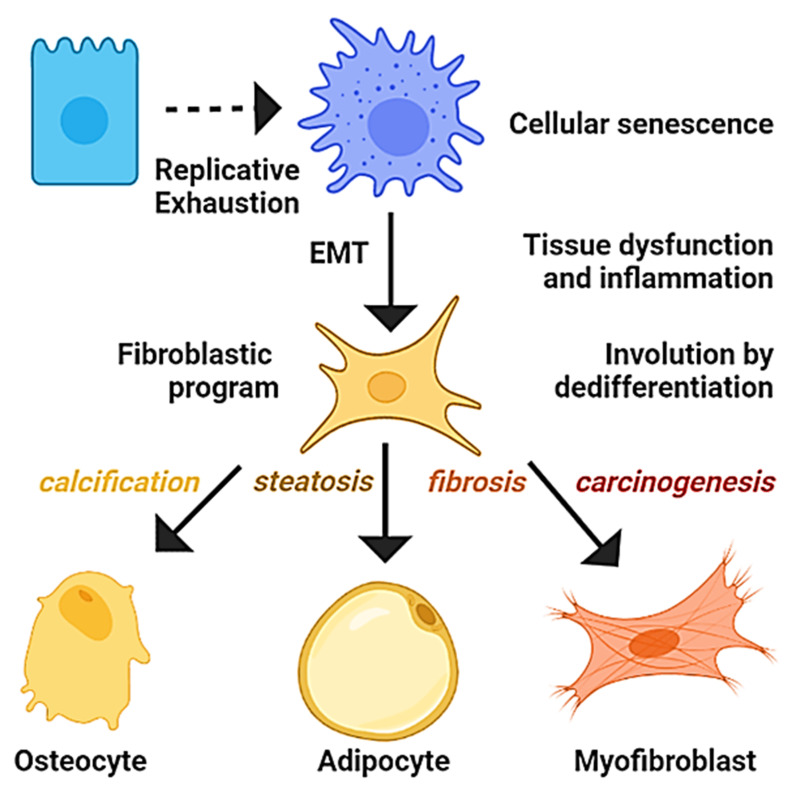
The hypothesis proposes to understand chronic diseases as the result of tissue dedifferentiation initiated by cellular senescence. The molecular and cellular events reviewed for epithelial carcinogenesis also appear to be involved in the susceptibility of aged cells to dedifferentiate into mesenchymal lineages and to explain the tendency of organs for calcification, steatosis, and fibrosis once they experience replicative exhaustion and inflammation. Those processes might underlie the observed dysfunction and the increased tendency of tissues for malignant transformation associated with aging.

Developmental biology studies the mechanisms that govern the development of organisms from fertilization to senescence. Aging is accompanied by a gradual decline in the biological functions associated with replicative exhaustion and organ involution, characterized by calcification, steatosis, and fibrosis [233]. Interestingly, fibroblasts derived from the process of EMT possess the potential to differentiate into osteocytes or adipocytes [22], and calcifications, fatty change, or the fibrosis of tissues have been related to the activities of osteogenic, adipogenic, and fibrogenic transcription factors, respectively, suggesting a process of dedifferentiation [234,235,236]. Importantly, progressive organ fibrosis is a major cause of morbidity and mortality, and the therapeutic options are handicapped by an incomplete understanding of its origin [237]. In addition, accumulating evidence attributes the fibrosis of vessels, heart, liver, kidney, and pancreas to EMT or related events of transdifferentiation [238,239]. In similitude to cancer, despite the current multitarget approaches to cope with cardiovascular diseases and diabetes *mellitus*, they remain the principal causes of morbidity and mortality among adults in industrialized countries [240]. Similarly, cellular senescence is associated with the functional cellular decline observed in chronic diseases, such as the inability of senescent vascular endothelial cells to generate nitric oxide and regulate the coronary vascular tone associated with hypertension [241], whereas the defective insulin secretion in senescent pancreatic beta cells impairs the regulation of glucose levels in diabetes *mellitus* [242]. Given the revised mechanisms, it is theorized that most chronic diseases are the result of the functional impairment of organs due to degeneration driven by replicative cellular senescence and inflammation, which promotes events of transdifferentiation into mesenchymal lineages (Figure 4).

Many aspects of the hypothesis should apply to well-known models of carcinogenesis. In this regard, changes in the cellularity during the gradual progression of the nonalcoholic fatty liver disease (NAFLD) seem to support the generic order of events and their proposed temporal occurrence. Although the detailed mechanisms responsible for the entire process of liver degeneration remain to be elucidated, recent discoveries have uncovered the chronological appearance of senescent, mesenchymal, and malignant stem cells as well as the potential involvement of the immune system in the process of transdifferentiation, thereby providing evidence for the origin of cancer cells and clues for the underlying mechanisms of the progressive degeneration observed in the NAFLD. In the following, some recent evidence consistent with the notion that hepatocytes become prone to dedifferentiate in response to stress or replicative exhaustion is revised.

The most common form of chronic liver disease in obese patients is the NAFLD [243]. Long-term exposure to stressful conditions can induce its progression from simple steatosis to advanced fibrosis, cirrhosis, or hepatocellular carcinoma [244,245]. Interestingly, the incidence of liver calcifications, steatosis, fibrosis, and carcinoma can co-occur and increases with age and inflammatory conditions [47,245,246]. It has been proposed that aberrant activation of the hepatic stem cells is involved in the genesis of those conditions since they might give rise to cancer cells [247], adipocytes, osteocytes, and fibroblasts [248]. The normal human adult liver contains progenitor cells that express embryonic markers, such as Oct, Nanog, Sox2, and the mesenchymal proteins vimentin, cytokeratin 8, and 18 together with albumin and α-fetoprotein indicating a mesenchymal origin with a partial commitment toward a hepatic lineage. In addition, the non-parenchymal fraction harbors another stem cell population that expresses the mesenchymal stem cell markers with hepatic proteins [248]. However, the fact that liver tumors arise from nodules [82] that present senescent cells [47,79] supports the notion that hepatocellular carcinoma emerges due to the dedifferentiation of mature hepatocytes.

The induction of hepatocellular carcinoma by diethylnitrosamine in mice produces hyperplasic lesions that expressed higher levels of α-fetoprotein and presented senescent hepatocytes with nuclear atypia and increased DNA content. Interestingly, the treatment with aflatoxin, carbon tetrachloride, or a high-fat diet resulted in similar observations [79]. Strikingly, the senescent hepatocytes display hyperpolyploidy and stemness and were able to induce tumors in immunodeficient mice. Noteworthily, the malignant cells derived from the senescent hepatocytes became small, and their subsequent treatment with diethylnitrosamine in vitro increased the content of cells co-expressing stem cell markers and vimentin. These observations led to the conclusion that hyperpolyploid hepatocytes acquire genome instability, and hence, tumorigenic behavior [79].

The study seems to overlook the phenotypical changes the senescent cells undergo in order to become stem and malignant. The hypothesis suggests that this finding corresponds with the transdifferentiation of senescent cells into malignant myofibroblasts by the process of EMT.

Supporting this statement, in a similar experiment after 10 weeks of diethylnitrosamine treatment, mice developed liver nodules with nuclear atypia and fibrosis. Fortunately, in this case, the liver sections were double-stained for β-gal and α-SMA and revealed that the higher levels of both biomarkers were present in the myofibroblast-like cells. In addition, the controls were negative for the presence of those cells, whereas the tumor showed a seven-fold increment at week 16. Other interesting findings were the enrichment of senescent myofibroblasts in the regions more exposed to diethylnitrosamine, their correspondence with the phenotype of the activated hepatic stellate cells (central mediators of liver fibrogenesis), and the overexpression of glutamyltranspeptidase (along with α-SMA found in the cancer-associated fibroblasts) in tumors surrounded by senescent cells [47].

According to the hypothesis, those results indicate that senescent hepatocytes transdifferentiate in vivo into myofibroblasts that give rise to hepatic stellate cells, cancer-associated fibroblasts, or epithelial tumors cells in accordance with their microenvironmental cues.

During the liver fibrosis induced by the administration of carbon tetrachloride, the immune cells migrate into the fibrotic scar creating an inflammatory environment adjacent to hepatic stellate cells with the senescent markers [86]. That finding was interpreted as the antitumoral activities of the immune system; contrastingly, in patients, the infiltration of the hyperplasic nodules is associated with proliferation and stemness in response to cytokines [82]. Monocytes are recruited to drive fibrosis and cirrhosis during chronic inflammation of the liver [249]. In tumor samples of patients with hepatocellular carcinoma, monocytes accumulate near the peritumoral activated hepatic stellate cells that increase the expression of inflammatory genes that correlate with poor prognosis and biomarkers of cancer stem cells [250]. Further studies show that the co-culture of macrophages with liver cancer cells resulted in the enhanced expression of stem and mesenchymal transcription factors with an increased sphere-forming capacity [154]. In addition, the hepatocytes isolated from cirrhotic livers display elongated fibroblastoid morphology and express vimentin [251]. Lineage tracing studies revealed that hepatocytes undergo EMT during fibrosis and acquire a fibroblast-like morphology with the expression of fibroblast-specific protein 1 (FSP-1) [252]. Furthermore, the inhibition of TGF-β prevented the carbon tetrachloride-induced liver fibrosis, suggesting that cytokines promote the emergence of fibroblasts by induction of EMT [253]. In turn, the process of EMT has been linked with the acquisition of the cancer stem cell phenotype and the emergence of chemoresistance in patients diagnosed with hepatocellular carcinoma [254,255].

The hypothesis provides an alternative interpretation of those findings in which immune cells are attracted to senescent hepatocytes and promote their dedifferentiation by EMT and access into a myofibroblastic undifferentiated state. Hence, these processes may underly the observation of stromal malignant cells produced in inflammatory conditions that stain positive for β-gal, α-SMA, and α-fetoprotein.

The influence of senescence and EMT in hepatic steatosis and calcifications remains largely unexplored. However, a recent report found a close relationship between the senescence markers, immune infiltration, and fat accumulation in hepatocytes of mice fed ad libitum. In addition, the ingestion of high-fat diets increased the senescent markers and the severity of steatosis [245]. The observation was attributed to mitochondrial dysfunction and impaired lipid metabolism; however, the liver steatosis seems also to be driven by the activities of the peroxisome proliferator-activated receptor-gamma (PPAR-γ) [256,257]. Fats are natural ligands of PPAR-γ; notably, the pharmacological overexpression of this protein induces the adipogenic program in myofibroblasts [258] and exacerbates fatty liver disease in obese mice [259]. Furthermore, the severity of steatosis has been associated with PPAR-γ dependent transcriptional responses leading to the possibility of hepatocyte–adipocyte transdifferentiation [234]. Interestingly, prolonged exposure of hepatocarcinoma cells to fatty acids in vitro and a high-fat diet in mice produce inflammation, an increased level of TGF-β, the mesenchymal transcription factors, and epithelial dedifferentiation. Hence, fats are involved in liver fibrosis induce the process of EMT [260]. Interestingly, recent discoveries in fibroblasts suggest that fats are actively participating in the determination of cell states by their influence on the activity of signaling receptors and the transcriptome [261].

To our knowledge, the potential role of hepatocyte dedifferentiation into osteocytes remains unexplained. However, evidence from other tissues suggests that it might also occur during liver degeneration. For example, in the case of the vascular smooth muscle cells, cellular senescence induces osteoblastic transition [235], whereas tumor calcifications express bone-related proteins [262].

The hypothesis proposes that aged hepatocytes are susceptible to epigenetic switches, but their cellular fate is influenced by microenvironmental signals. Diets, metabolic conditions, or drugs that increase the availability of PPAR-γ ligands could contribute to the emergence of adipocytes in the stressed liver. In the case of the osteocytes, the increment in the molecular damage, infiltration, and inflammation might promote the activation of the osteogenic program, explaining their appearance in tumors. In summary, it is plausible that aged hepatocytes that undergo EMT by the influence of the immune system became a potential source for osteocytes, adipocytes, fibroblast, myofibroblast, and cancer stem cells. Hence, the progression of the NAFLD can be understood as a process emerging from dedifferentiation.

The reviewed reports highlight that cellular senescence and inflammation are mechanistically connected with discrete changes in the cellular phenotype and may potentially underly the degeneration or the malignant transformation of tissues associated with aging and its chronic diseases. Altogether, it seems plausible that terminal epithelial cells that experience cellular senescence and EMT are allowed to reiterate cellular states in their trajectory of differentiation. Similarly, the recovery of cellular plasticity enables the adoption of mesenchymal-stem programs to cope with stress. All of this would explain the reactivation of developmental programs within neoplasia, the correspondence of cancer subtypes with normal cell states that compose the tissues, as well as the link of senescence, inflammation, and EMT with the emergence of resistance. Overall, the hypothesis suggests that genome instability derived from replicative cellular senescence induces plasticity, which manifests as dedifferentiation that underlies the susceptibility of epithelial cells to degenerate into stromal lineages during aging.

## 6. Conclusions

The definitive mechanistic basis of the connection between DNA damage, aging, inflammation, and the origin of chronic diseases remains unclear. Understanding the fundamental causes for their emergence has the potential to reshape our methods of detection, prevention, and management. In this regard, proposing novel and more encompassing theoretical frameworks for the study of degenerative diseases may foster insights and experiments that lead to advances in our knowledge. Hence, this article provides an alternative explanation for the conserved pattern of the molecular and cellular events observed during the histological progression of carcinomas and the rationale behind the reiterative emergence of the cellular phenotypes involved in the process. In addition, the hypothesis presented for the origin of carcinomas intuitively provides a plausible mechanistic explanation for the calcification, steatosis, and fibrosis that characterizes the degeneration, the loss of function, and, in some cases, the transformation of tissues during aging from a molecular perspective. In summary, it is postulated that cellular senescence will precede the onset of most chronic diseases. Then, inflammation and the infiltration of the affected tissues may accelerate their degeneration by increasing their chances of dedifferentiation. In this context, the emergence of mesenchymal phenotypes will be promoted by telomere attrition or tissue disruption, leading to parenchyma degeneration that results in its substitution with stromal content. It can be considered that the adoption of mesenchymal lineages reflects the behavior of cells with telomere attrition in a milieu defective in normal parenchyma, stroma, hormones, and growth factors, since epithelial cells are highly dependent on ligands produced by other specialized cells and are fine-tuned by structural interactions. Therefore, aging implies the adoption of mesenchymal and more robust cellular states that are deprived of normal functions and manifest with the onset of chronic diseases. A logical consequence of the hypothesis implies that conditions promoting and sustaining the emergence of myofibroblasts will foster malignant transformation of the tissues since cells in that state are endowed with plasticity and primed for tumorigenesis and metastatic behavior. Accordingly, any attempt to cure cancer that involves the induction of senescence, inflammation, or EMT would result in a temporal remedy followed by organ fibrosis, the worsening or the beginning of chronic diseases, and the eventual emergence of tumors with increased malignancy. In conclusion, the degeneration of tissues seems to originate from the loss of genomic stability due to replicative exhaustion, which triggers the emergence of more robust but nonfunctional mesenchymal cells. Much remains to be understood in terms of scientific knowledge, but the possible molecular link of most chronic diseases, in which the same conserved events govern their pathogenesis, may spur the development of novel and integrative therapeutic approaches.

## Data Availability

Not applicable.
